# Modeling and Analysis of Data-Driven Systems through Computational Neuroscience Wavelet-Deep Optimized Model for Nonlinear Multicomponent Data Forecasting

**DOI:** 10.1155/2021/8810046

**Published:** 2021-06-12

**Authors:** Xue-Bo Jin, Jia-Hui Zhang, Ting-Li Su, Yu-Ting Bai, Jian-Lei Kong, Xiao-Yi Wang

**Affiliations:** ^1^School of Artificial Intelligence, Beijing Technology and Business University, Beijing 100048, China; ^2^China Light Industry Key Laboratory of Industrial Internet and Big Data, Beijing Technology and Business University, Beijing 100048, China; ^3^Beijing Key Laboratory of Big Data Technology for Food Safety, Beijing Technology and Business University, Beijing 100048, China

## Abstract

Complex time series data exists widely in actual systems, and its forecasting has great practical significance. Simultaneously, the classical linear model cannot obtain satisfactory performance due to nonlinearity and multicomponent characteristics. Based on the data-driven mechanism, this paper proposes a deep learning method coupled with Bayesian optimization based on wavelet decomposition to model the time series data and forecasting its trend. Firstly, the data is decomposed by wavelet transform to reduce the complexity of the time series data. The Gated Recurrent Unit (GRU) network is trained as a submodel for each decomposition component. The hyperparameters of wavelet decomposition and each submodel are optimized with Bayesian sequence model-based optimization (SMBO) to develop the modeling accuracy. Finally, the results of all submodels are added to obtain forecasting results. The PM2.5 data collected by the US Air Quality Monitoring Station is used for experiments. By comparing with other networks, it can be found that the proposed method outperforms well in the multisteps forecasting task for the complex time series.

## 1. Introduction

Usually, the data we collect in the existing system is complex time-series data, such as air pollution data [1], i.e., PM2.5, PM10, and O3. The forecasting of these pollution content is essential for air quality control. As to the PM2.5 forecasting problem, accurate multisteps forecasting is more meaningful because it can provide faster response time to control and manage air quality. The data at each moment is the value of the last moment that changes over time and is affected by factors such as weather, industrial production, and people's lives. Due to the multicomponent and nonlinearity of the data, the forecasting research is still an open issue, especially for multistep forecasting.

The classical method, probability methods [2], is limited by the prior given knowledge. If the assumed model does not match the actual data distribution, it often fails to provide a correct forecasting result. Therefore, mechanism-based modeling is challenging for PM2.5 data.

On the other hand, the data-driven learning method [3] is more adaptable for modeling based on the historical data without requiring prior knowledge. Therefore, data-driven learning methods, such as the deep learning method, perform better in nonlinear complex dynamic forecasting tasks. Thus, in recent years, data-driven modeling methods have shown significant advantages in PM2.5 modeling and forecasting.

However, due to the complexity, limited amount, and the data's incompleteness, we found that the deep learning network forecasting results still need to be improved, especially for multistep prediction. The data-driven learning methods are often implemented through the iterative schemes [4–8] and the recursive schemes [9–13], including the recursive least squares algorithms [14–18] and the gradient-based search algorithms [19–23].

In this paper, a data-driven model is proposed to the multisteps ahead forecast.  Section 2 discusses the related work of time series modeling and forecasting and analyzes probability and learning method's advantages and disadvantages. Then, Section 3 gives the proposed model's details, including the decomposition by wavelet transform, the Gated Recurrent Unit (GRU) as a submodel, and Bayesian optimization for the hyperparameters. As a practical example, the experiment based on the Beijing PM2.5 is conducted to improve the proposed model. The results of 2 cases are shown in Section 4. Finally, the conclusions are discussed in Section 5.

## 2. Related Works

Probability methods [24, 25], such as ARIMA, dynamic regression model, and the autoregressive threshold model, are quite challenging to get accurate model due to the difficulty of obtaining the prior knowledge required. While learning methods, such as the linear regression forecasting model [26–28], can get the hidden relationship between the data through adaptive learning.

With the depth of time series forecasting research, the shallow network based on artificial neural network (ANN) has been used to solve the nonlinear time series forecasting problem [29–31]. Ye et al. proposed a self-applicable BP neural network, which established the relationship between the aerosol optical depth and the PM2.5 data [32]. Bai et al. gave a method combined with the autoregressive network and BP network for nonlinear data modeling [33]. However, due to the limitation of the network depth, the network cannot accurately model the complex data for accurate multisteps forecasting.

Recently, the emergence of recurrent neural network (RNN) and its higher accuracy in nonlinear time series forecasting tasks have attracted many researchers' attention. For example, the RNNs are used for the forecasting of PM10 and PM2.5 [34]. However, due to the RNN network structure's limitations, the effect will be worse for multistep forecasting. The emergence of long short-term memory (LSTM) solves the multisteps dependency problem of RNN [35, 36]. Unlike the LSTM, the Gated Recurrent Unit (GRU) further simplifies the composition of the LSTM while maintaining the accuracy of the forecasting [37]. For these deep networks, the hyperparameters determine the performance of the model. However, the hyperparameters selected randomly have resulted in lower performance for modeling.

On the other hand, due to the complex dynamical nonlinearity and multiple components with different frequencies [38], deep learning networks' PM2.5 forecasting performance still needs to be improved, especially multistep forecasting. Therefore, the data decomposition is added to the forecasting model, and it turns out that this method can indeed improve the accuracy of forecasting.

As one of the decomposition methods, seasonal trend decomposition procedure based on loess (STL) [39–41] can obtain trend, seasonal, and residual components of complex data. Similarly, empirical mode decomposition (EMD) [42–44] is also often used to analyze time-series data with higher complexity. EMD decomposes a time series into multiple mode functions (IMF), which reflect the frequency differences of the original data. In our previous research [45], we propose a multistep forecasting model for atmospheric PM2.5 concentration based on EMD decomposition. The obtained IMF components were divided into three groups according to their frequency characteristics. Also, the integrated empirical mode decomposition (EEMD) method is used very frequently. It is an improvement of the EMD method. The modal aliasing problem of the EMD method is solved. Similar to the EMD method, it decomposes the time series into multimodal functions. Nguyen et al. proposed a self-enhancement mechanism based on the EEMD method [46], which decomposes the time series into multiple intrinsic mode functions and divides the intrinsic mode into a strong and a weak correlation part by K-means. These two parts are used for multitask learning and multiview learning, respectively. Finally, the result is obtained through fusion.

The decomposition methods have been used in many areas, such as signal processing and system identification. Many state estimation and parameter identification algorithms have been proposed for linear systems [47–49], bilinear systems [50–55], and nonlinear systems [56–58]; its basic idea is the hierarchical identification principle. These methods can be used for modeling and prediction of time series. Unlike the above decomposition methods, wavelet decomposition [59] can choose an appropriate mother wavelet function to decompose one-dimensional information into multidimensional information. It can set the number of decomposition layers, which means that the number of components is controllable. Wavelet decomposition has an excellent performance in processing multiscale information and can transform the signal into two parts: low frequency and high frequency. Each frequency is independent of the other. Cheng et al. proposed combining wavelet decomposition with traditional forecasting models (including ANN, ARIMA, and SVM) and proposed three hybrid models for short-term PM2.5 forecasting [60]. Wang et al. proposed a forecasting network combining wavelet decomposition and LSTM network to forecast solar radiation intensity in different weather environments and compare it with traditional and single deep learning networks [61].

In this paper, we propose the model with a wavelet decomposition, the GRUs group (WD-GRU) based on the Bayesian optimization for the hyperparameters, and forecast the multisteps for the Beijing PM2.5 data. Our contributions focus on the following:

1. The proposed model utilizes the hyperparameters optimization of the whole model to improve performance. Sequence model-based optimization (SMBO) is utilized to optimize the hyperparameters, including the number of wavelet layers, the type of mother wavelet function, the number of neurons in the first layer of GRU, epoch, the dropout rate, batch-size, and the type of optimizer.
2. The WD-GRU model is proposed, in which wavelet decomposition is used to decompose the original data to reduce the complexity of the time series data. Then each component is forecasted separately by GRU, and the result is finally obtained by fusion. Compared with the model with WD-LSTM [61], the model proposed here improves the forecasting performance for the application of PM2.5.

## 3. Deep Model

The model proposed here is a model with a combined structure in which wavelet decomposition is used to reduce the data's nonlinear complexity. GRUs are used for each component to forecast, and the final forecasting will be obtained according to each submodel's results. And the hyperparameters of the whole model are optimized through SMBO. We will describe each part of the proposed model in detail below.

### 3.1. Decomposition of Time Series Data

In this section, we decompose the time series data into a limited number of low-frequency subsequences and high-frequency subsequences according to time series data characteristics. The discrete wavelet transform (DWT) algorithm is used to achieve the above process. It can be found that the subsequence obtained after decomposition has a more stable variance than the original sequence. It can reduce the complexity of data, which helps increase the forecasting performance of the time series.

In numerical analysis, the DWT is derived from the Fourier transform, while the DWT uses the different basic functions, i.e., not the infinite triangular bases, but the finite-length and decaying wavelet bases are used. DWT need to specify a mother wavelet function *η*(*t*), such as “db35”; after translation and amplification of *η*(*t*), the corresponding function *η*_*k*,*h*_(*t*) is obtained by

(1) $$\begin{array}{c} \eta_{k,h}\left(t\right)=2^{k/2}\eta \left(2^{k}t-h\right). \end{array}$$

Moreover, we can calculate the corresponding binary function *ψ*_*k*,*h*_(*t*):

(2) $$\begin{array}{c} \psi_{k,h}\left(t\right)=2^{k/2}\psi \left(2^{k}t-h\right), \end{array}$$

where *k* is the scaling factor and *k* ∈ *R*; *k* ≠ 0; *h* is the translation factor and *h* ∈ *R* , and *t* is the time index. In the DWT process, *η*_*k*,*h*_(*t*) and *ψ*_*k*,*h*_(*t*) are called wavelet bases. For a time series data *M*(*t*), the DWT algorithm can be expressed as

(3) $$\begin{array}{c} M\left(t\right)=\sum_{h=1}^{m}a_{k,h}\psi_{k,h}\left(t\right)+\sum_{k=1}^{n}\sum_{h=1}^{m}d_{k,h}\eta_{k,h}\left(t\right), \end{array}$$

where *a*_*k*,*h*_ is the low-frequency component with a scaling factor of *k* and a translation factor of *h* and *d*_*k*,*h*_ is the high-frequency component with a scaling factor of *k* and a translation factor of *h*. *m* is the length of the original time series data. *n* is the number of layers of the wavelet decomposition. So DWT can decompose time series into low-frequency subsequences and high-frequency subsequences. Then a low-pass filter (LPF) and a high-pass filter (HPF) are used to obtain low-frequency subsequence *A*_*k*,*h*_ and high-frequency subsequence *D*_*k*,*h*_ based on *a*_*k*,*h*_ and *d*_*k*,*h*_.

**Figure 1:**
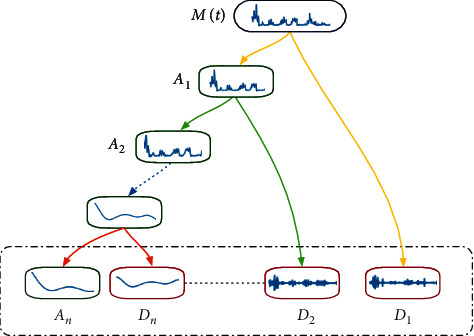
Schematic of the wavelet decomposition process.

Figure 1 shows the wavelet decomposition process in the actual decomposition task, assuming that *M*(*t*) is the time series being decomposed. In the first layer of the wavelet decomposition space, the time series *M*(*t*) is decomposed into a low-frequency subsequence *A*_1_ and a high-frequency subsequence *D*_1_. We have the process of the DWT as

(4) $$\begin{array}{c} \eta_{1,h}\left(t\right)=2^{1/2}\eta \left(2t-h\right),\\ \psi_{1,h}\left(t\right)=2^{1/2}\psi \left(2t-h\right),\\ M\left(t\right)=\sum_{h=1}^{m}a_{1,h}\psi_{1,h}\left(t\right)+\sum_{h=1}^{m}d_{1,h}\eta_{1,h}\left(t\right), \end{array}$$

where *A*_1_ is the result of *a*_1,*h*_ with the length *m* through LPF and *D*_1_ is the result of *d*_1,*h*_ with the length *m* through HPF. Then according to the defined number of decomposition layers, the approximate subsequence will continue to decompose according to the decomposition rules, the low-frequency subsequence *A*_1_ continues to decompose for *A*_2_ and *D*_2_, and so on. That is to say, for the time series *M*(*t*), after the *n* layer decomposition, the set of {*A*_*n*_, *D*_1_, *D*_2_,…*D*_*n*_} is finally obtained, and there is a relationship

(5) $$\begin{array}{c} M\left(t\right)=A_{n}+D_{1}+D_{2}+\cdots +D_{n}. \end{array}$$

To further analyze wavelet decomposition, we take the 100-day Beijing PM2.5 hourly data from January 1, 2016, as an example to perform wavelet decomposition, and the length of the decomposed discrete sequence is 2,400 hours.

**Figure 2:**
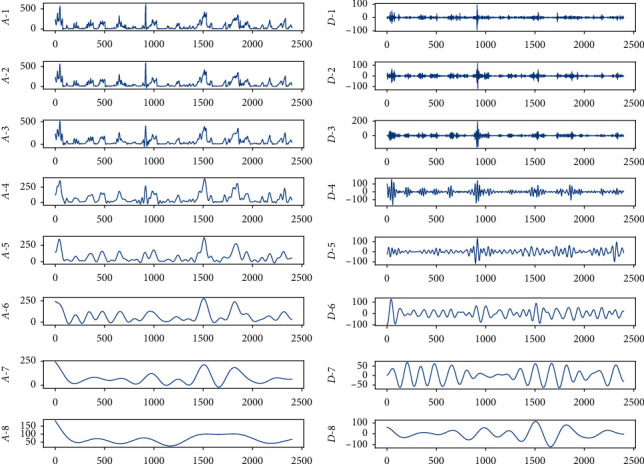
8-layer decomposition results using db35. Left to right: (a) low-frequency components and (b) high-frequency components. In the first layer, *M*(*t*) is decomposed to the line *A*-1 and the line *D*-1, and then *A*-1 is decomposed to the line *A*-2 and the line *D*-2, until the last layer, i.e., the eighth layer; *A*-7 is decomposed to the line *A*-8 and the line *D*-8.

The db35 mother wavelet function is used, and the number of decomposition levels is 8.  Figure 2(a) shows the average low-frequency results of each layer decomposition, and Figure 2(b) shows the high-frequency components of each layer decomposition. We can see that the low-frequency and high-frequency components have apparent changes as the number of decomposition layers increases, and the lines are gradually flat, which shows that the wavelet decomposition successfully decomposes a complex sequence into several subsequences with a single frequency.

For an actual signal, the number of layers *n* is determined by its length. A signal with the length *m* can only be decomposed into log_2_  *m* layers at most. There is not a standard principle to select the level of decomposition layers and the mother wavelet function. In contrast, they will determine the decomposition result, and further have an effect on the forecasting performance. Therefore, we will use Bayesian optimization to determine the type of the mother wavelet function and decomposition layers of our model, which will be discussed in Section 3.3.

### 3.2. Deep Submodel for Wavelet Decomposition Components

To model each component, we use the GRU network, which is an improvement in LSTM. Each neuron in the network is a processing unit that includes an update gate and a reset gate. The update gate is to replace the previous state information with the current state. The reset gate controls the degree of ignoring the last information status, and the GRU unit has only one timing output.

The calculation formula in each unit when performing forward propagation according to this structure is as follows [62–64]:

(6) $$\begin{array}{c} z_{i}=\sigma \left(C_{z}x_{i}+y_{i-1}U_{z}+b_{z}\right),\\ r_{i}=\sigma \left(C_{r}x_{i}+y_{i-1}U_{r}+b_{r}\right),\\ a_{i}=\tanh\left(C_{a}x_{i}+U_{a}\left(y_{i-1}*r_{i}\right)+b_{a}\right),\\ y_{i}=\left(1-z_{i}\right)*y_{i-1}+z_{i}*a_{i}, \end{array}$$

where *σ* is the Sigmoid activation function, *x*_*i*_ represents the input at the time *i*, *z*_*i*_ is the attenuation coefficient of the updaters, *r*_*i*_ is the attenuation coefficient of the reset gate, *y*_*i*−1_ is the output value at the time *i* − 1, *y*_*i*_ is the output state vector at a time *i*, *C*_*z*_ and *U*_*z*_ are the weights of the update gate, *C*_*r*_ and *U*_*r*_ are the weights of the reset gate, *C*_*a*_ and *U*_*a*_ are the weights of the candidate *a*_*i*_, *b*_*z*_, *b*_*r*_, and *b*_*a*_ are offset vectors, and *∗* is an element-wise multiplication.

**Figure 3:**
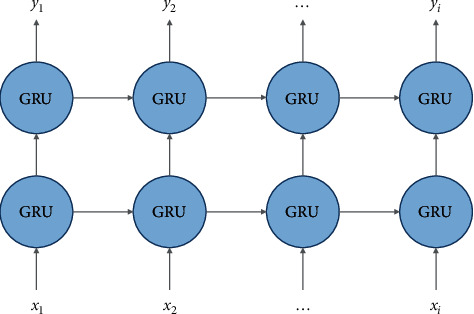
GRU network structure for the submodel.

The hidden layer of the GRU network is set to 2 layers, and the activation function is “relu.” To prevent the training network from overfitting, we added the dropout in each layer.  Figure 3 shows the construction of each submodel, where *x*_*i*_, *i*=1,2,…, *m* is the input and *y*_*i*_, *i*=1,2,…, *m* is the output.

The model training uses the *L*_1_ loss function, which can obtain better robustness for forecasting the time series data with noise such as PM 2.5. The *L*_1_ loss function is selected as

(7) $$\begin{array}{c} L_{1}\left(\hat{y},y,\theta \right)=\sum_{i=1}^{m}\left|y_{i}\left(\theta \right)-{\hat{y}}_{i}\right|, \end{array}$$

where *θ* is the weight of the network, *y* is the forecasting result, and $\hat{y}$ is the ground truth.

Thanks to deep learning research, there are many ways to update deep networks' weights based on the loss function, such as Adadelta, Adam, and Sgd. And the other hyperparameters, such as dropout rate, batch-size, and the number of epochs, will also affect the capability of deep learning networks. To guarantee performance, we will use the Bayesian SMBO method to select these hyperparameters. Not only can WD-GRU be used for air quality monitoring research but it also forms a new network by combining with other networks, which can be used in other research fields, such as the research on prediction and management control of water environment [65–67] and IoT intelligence [68].

### 3.3. Bayesian Sequence Model-Based Optimization (SMBO)

Hyperparameters are one of the keys to deep learning models, directly determining the performance of the model. Due to the deepening of the forecasting model network, the selection of hyperparameters becomes a difficult problem. But, the traditional method of selecting parameters is inefficient. It cannot be used at all when there are too many hyperparameters, so the chosen hyperparameters are also challenging to keep the model perform well. Here, we use the Bayesian SMBO algorithm [69, 70] to optimize the hyperparameters, including the hyperparameters of the deep learning model and wavelet decomposition as the number of decomposition layers.

For SMBO, the key is to give an optimization objective function. In the parameter space, the Gaussian process is used to update the posterior distribution of the objective function to seek a group parameter that maximizes the objective function. The RMSE is used as the objective function:

(8) $$\begin{array}{c} g\left(w\right)=\sqrt{\frac{{\sum_{i=1}^{m}\left(y_{i}\left(w\right)-{\hat{y}}_{i}\right)}^{2}}{m}}, \end{array}$$

where *m* is the length of the input series, *y*_*i*_(*w*) is the forecasting result by the hyperparameters *w*, and ${\hat{y}}_{i}$ is the ground truth. The objective function of SMBO is minimized as

(9) $$\begin{array}{c} w^{*}=\underset{w\in W}{\arg\min}g\left(w\right), \end{array}$$

where *w*^*∗*^ is the optimal parameter determined by SMBO; *w* is a set of input hyperparameters, including not only the weight of the network *θ*, but also the mother wavelet functions and the level of decomposition layers. *W* is the multidimensional hyperparameters space defined for the optimized model.

The SMBO algorithm can generally be divided into two processes: Gaussian process and hyperparameter selection. In the Gaussian process, the modeling and fitting optimization of the objective function is achieved, and the posterior distribution corresponding to the input *w* is obtained; in the hyperparameter selection process, the optimal hyperparameters are explored at the minimum cost. According to the objective function *g*(*w*), we set the Gaussian distribution as follows:

(10) $$\begin{array}{c} g\left(w\right)∼GP\left(\mu \left(w\right),O\left(w,w\prime \right)\right), \end{array}$$

where *μ*(*w*) is the average value of *g*(*w*) and *O*(*w*, *w*′) is the covariance matrix of *g*(*w*). The initial *O*(*w*, *w*′) can be expressed as

(11) $$\begin{array}{c} O=\left[\begin{array}{ccc} o\left(w_{1},w_{1}\right) & \cdots & o\left(w_{1},w_{i}\right)\\ \vdots & \ddots & \vdots \\ o\left(w_{i},w_{1}\right) & \cdots & o\left(w_{i},w_{i}\right) \end{array}\right]. \end{array}$$

In the process of SMBO searching for optimal parameters, the covariance matrix of the above Gaussian process will continuously change during the iterative process. Assuming that the set of parameters entered in step *i*+1 is *w*_*i*+1_, then the covariance matrix is

(12) $$\begin{array}{c} O^{\prime }=\left[\begin{array}{cc} O & o^{T}\\ o & o\left(w_{i+1},w_{i+1}\right) \end{array}\right], \end{array}$$

where *o*=[*o*(*w*_*i*+1_, *w*_1_), *o*(*w*_*i*+1_, *w*_2_),…, *o*(*w*_*i*+1_, *w*_*i*_)]. Then we get the posterior probability of *g*(*w*) as

(13) $$\begin{array}{c} P\left(g_{i+1}|D_{i+1},w_{i+1}\right)∼N\left(\mu_{i+1}\left(w\right),\sigma_{i+1}^{2}\left(w\right)\right), \end{array}$$

where *D* is the observation data, *μ*_*i*+1_(*w*) is the mean value of *g*(*w*) at step *i*+1, and *σ*_*i*+1_^2^(*w*) is the variance of *g*(*w*) at step *i*+1.

After obtaining the posterior probability, the next step is to find the optimal parameters through hyperparameter selection. This search method is complicated and takes a lot of time, so we use the following upper confidence bound (UCB) acquisition function to develop the calculation effectiveness:

(14) $$\begin{array}{c} w_{i+1}=\arg\max H\left(w|D_{i}\right)=\arg\max\mu \left(w\right)+\zeta_{i+1}^{1/2}\sigma_{i}\left(w\right), \end{array}$$

where *ζ*_*i*+1_ is a constant, *H*(*w|D*_*i*_) is the UCB acquisition function, and *w*_*i*+1_ is the selected hyperparameter of step *i*+1. The SMBO algorithm of the network is shown in Algorithm 1.

### 3.4. Model Framework with the Optimization of Hyperparameters

**Figure 4:**
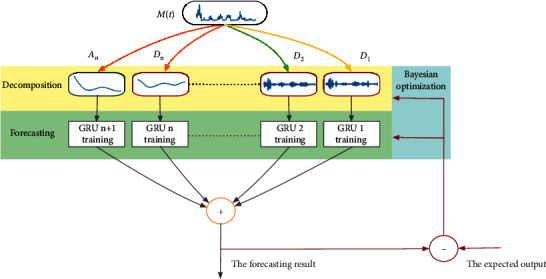
Flowchart of the model for forecasting.

Based on the details introduced in Section 3.1–Section 3.3, Figure 4 shows the proposed deep forecasting model. Firstly, the original time series is decomposed based on wavelet decomposition to obtain the corresponding low-frequency subsequences *A*_*n*_ and high-frequency subsequences *D*_1_, *D*_2_,… *D*_*n*_, and then GRU is trained to learn each component of dynamic characteristics. The trained GRU is then used to separately forecast the subsequences obtained by decomposition and finally achieve the forecasting.

During the model's training, the SMBO algorithm optimizes hyperparameters based on the forecasting result and the expected output. Once the optimized parameters have been obtained, the Bayesian optimization process will stop. Then the whole model is applied to the forecast.

## 4. Experiments

### 4.1. Dataset and Experimental Setup

The PM2.5 dataset of the US State Department [71] is used to verify the proposed model's effect, including the average PM2.5 concentration per hour in Beijing's atmosphere from 2013 to 2017, totaling 37,704 hours. The unit of the data is *μg*/*m*^3^. We use PM2.5 data to train our proposed model and other comparative models. The learning step is set to 24; that is, the model function is to use the data of 24 hours of the previous day to forecast the value of 24 hours of the next day. The forecast hourly of one day in advance is of great significance, which can help people understand the PM2.5 situation of the next day and plan the next day according to the numerical response's weather conditions.

It is often more reasonable to have enough performance evaluation indicators in the experimental verification stage. We use 5 indicators to assess the performance of our models, including root means square error (RMSE), normalized mean square error (NRMSE), mean absolute error (MAE), symmetric mean absolute percentage error (SMAPE), and Pearson correlation coefficient (*R*). The smaller the first four indicators are, the more accurate the forecasting is. *R* represents the Pearson correlation coefficient; the larger the value is, the closer the fitted relation between the ground truth and the forecasted value is. The calculation methods of 5 indicators are as follows:

(15) $$\begin{array}{c} \text{RMSE}=\sqrt{\frac{{\sum_{i=1}^{m}\left(y_{i}-{\hat{y}}_{i}\right)}^{2}}{m}}, \end{array}$$

(16) $$\begin{array}{c} \text{NRMSE}=\frac{1}{\max\left(y\right)-\min\left(y\right)}\sqrt{\frac{{\sum_{i=1}^{m}\left(y_{i}-{\hat{y}}_{i}\right)}^{2}}{m},} \end{array}$$

(17) $$\begin{array}{c} \text{MAE}=\frac{1}{m}\sum_{i=1}^{m}\left|y_{i}-{\hat{y}}_{i}\right|, \end{array}$$

(18) $$\begin{array}{c} \text{SMAPE}=\frac{1}{m}\sum_{i=1}^{m}\frac{\left|{\hat{y}}_{i}-y_{i}\right|}{\left(\left|{\hat{y}}_{i}\right|+\left|y_{i}\right|\right)/2}, \end{array}$$

(19) $$\begin{array}{c} R=\frac{\sum_{i=1}^{m}\left({\hat{y}}_{i}-{\bar{\hat{y}}}_{i}\right)\left(y_{i}-{\bar{y}}_{i}\right)}{\sqrt{\sum_{i=1}^{m}{\left({\hat{y}}_{i}-{\bar{\hat{y}}}_{i}\right)}^{2}\sum_{i=1}^{m}{\left(y_{i}-{\bar{y}}_{i}\right)}^{2}}}, \end{array}$$

where *m* is the length of datasets, $\hat{y}$ is the ground truth, *y* is the forecasting result, max(*y*) is the maximum of *y*, min(*y*) is the minimum of *y*, $\bar{\hat{y}}$ is the average of the ground truth, and $\bar{y}$ represents the average of a forecasted value.

Our experiment was conducted for the experimental platform using a PC server under Windows 10 operating system. The CPU is Intel (*R*) i5-6200U CPU, the single-core operating frequency is 2.30 GHz, and the RAM is 8 GB. Use *Python* 3.7.3 and Keras library to build the WD-GRU forecasting model, making the program more concise.

### 4.2. Case 1: Hyperparameter Selection Based on Bayesian Optimization

This case is based on the data set mentioned in Section 4.1, and the PM2.5 content per hour in Beijing from March 22, 2016, to April 9, 2016. The hourly content is forecasted, and the forecast period is 24 hours. In this case, we evaluate the hyperparameters of the WD-GRU forecasting model optimized by the Bayesian SMBO algorithm. To verify the SMBO algorithm's effectiveness in determining the number of wavelet decomposition layers and analyze the effect of decomposition layers on the model performance, we compare it with the traditional random search (random search) hyperparameter selection method.

Firstly, we define a multidimensional hyperparameter space for the WD-GRU model.  Table 1 shows the multidimensional hyperparameter space. The selected hyperparameters include decomposition layers, mother wavelet function, the number of neurons in the first layer, batch-size, epochs, optimizer, and dropout rate. Then we optimize the overall RMSE of the forecasting model; after 100 epochs, Bayesian optimization gives a set of optimal hyperparameters.  Table 2 shows the selected parameter set by the SMBO algorithm from the search space and the parameters chosen by the random search method in the common deep learning toolbox. We can find that there is obvious difference between the two sets of parameters.

We can note that the number of wavelet decomposition layers selected by SMBO is 8. And the wavelet function given by SMBO is db35. To verify it is reasonable, in this case, we conduct experiments on the effect of WD of different layers with the proposed model. This experiment uses the db35 mother wavelet function to decompose the PM2.5 sequence and then uses the two-layer GRU submodel mentioned in Section 3.2 for model training. The other hyperparameters of the submodel use SMBO parameters in Table 2. Then we test and verify the previously defined test set. The specific settings of the decomposition layers for the test model are as follows:

1. Mode no. 1: perform 1 layer of WD and train 2 GRUs for *A*_*1*_ and *D*_*1*_, respectively
2. Mode no. 2: perform 2 layers of WD and train 3 GRUs for *A*_*2*_, *D*_*1*_, and *D*_*2*_, respectively
3. Mode no. 3: perform 3 layers of WD and train 4 GRUs for *A*_*3*_, *D*_*1*_–*D*_*3*_, respectively
4. Mode no. 4: perform 4 layers of WD and train 5 GRUs for *A*_*4*_, *D*_*1*_–*D*_*4*_, respectively
5. Mode no. 5: perform 5 layers of WD and train 6 GRUs for *A*_*5*_, *D*_*1*_–*D*_*5*_, respectively
6. Mode no. 6: perform 6 layers of WD and train 7 GRUs for *A*_*6*_, *D*_*1*_–*D*_*6*_, respectively
7. Mode no. 7: perform 7 layers of WD and train 8 GRUs for *A*_*7*_, *D*_*1*_–*D*_*7*_, respectively
8. Mode no. 8: perform 8 layers of WD and train 9 GRUs for *A*_*8*_, *D*_*1*_–*D*_*8*_, respectively
9. Mode no. 9: perform 9 layers of WD and train 10 GRUs for *A*_*9*_, *D*_*1*_–*D*_*9*_, respectively
10. Mode no. 10: perform 10 layers of WD and train 11 GRUs for *A*_*10*,_*D*_*1*_–*D*_*10*_, respectively

Table 3 shows the forecasting results under different decomposition layers, where red is the best value, and the training of mode no. 8 uses the hyperparameters determined by the SMBO algorithm. We found that as the number of decomposition layers increases, these five indicators show an overall optimization trend. When the number of decomposition layers is set to 6, the value of RMSE has decreased from 48.5712 *μg*/m^3^ to 22.0185 *μg*/m^3^. Mode no. 8 obtains the least MAE and NRMSE as 16.2063 and 0.0682 and is very close to the optimal value of RMSE, SMAPE, and *R*.

Based on the above experimental results, we conclude that the number of wavelet decomposition layers determined by SMBO is the optimal solution in the hyperparameter space. Simultaneously, we find that the more layers the decomposition performs, the better the final model's forecasting effect is. When the number of decomposition layers reaches a specific value, the model's performance will no longer improve. If we continue to increase the number of decomposition layers, it will cause the model's overall performance to decline, for example, the mode no.10 with 10 levels. We analyzed this phenomenon and found that when too many decomposition layers are defined for data, false frequencies appear in the decomposition results. These are not the original signal's information, and this information leads to the deterioration of the forecasting results.

After learning the feasibility of the SMBO, to further explore the advantages of the SMBO algorithm, we then use the two sets of hyperparameters in Table 2 to train the WD-GRU model and conduct the test experiment.  Table 4 shows the performance indicators of the two models. We find that the model trained using the hyperparameters determined by the SMBO algorithm is significantly better. RMSE, MAE, NRMSE, SMAPE, and *R* increased by 4.9129 *μg*/m^3^, 2.5653 *μg*/m^3^, 0.0206 *μg*/m^3^, 0.0349, and 0.035, RMSE reached 21.7300 *μg*/m^3^, the *R* was higher than 0.9.

In summary, the SMBO algorithm is useful for selecting the hyperparameters of the proposed model. We verified its feasibility in the experiment of decomposing layers. And through comparing the model trained with the random search hyperparameter method, it is verified that the hyperparameter set determined by the SMBO algorithm can make the proposed model obtain a better forecast effect.

### 4.3. Case 2: Forecasting Performance Verification

To verify the WD-GRU model's performance advantages, we choose five combination models of decomposition method and deep learning methods to compare with the models proposed in this case. The comparison models used include decomposition-ARIMA-GRU-GRU [38], EMD_RNN [43] (EMD based on GRU), EMDCNN_GRU [45] (EMD and CNN-based on GRU), WD-RNN [34], and WD-LSTM [61].

**Figure 5:**
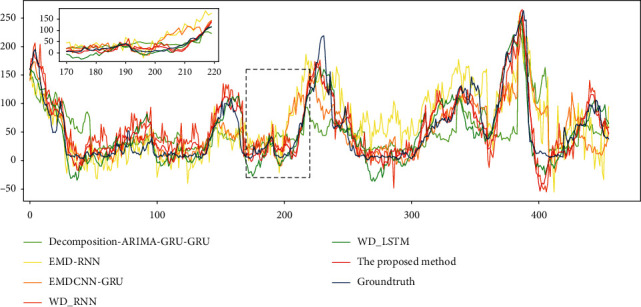
The forecasting of hourly PM2.5 in Beijing from March 22, 2016, to April 9, 2016, by decomposition-ARIMA-GRU-GRU [38], EMD-RNN [43], EMDCNN_GRU [45], WD-RNN [34], WD-LSTM [61], and the proposed method.

Figure 5 shows the forecasting trend curves of these six models. We use the red curve to represent the WD-GRU model proposed here. We can see that the WD-GRU model is closest to the ground truth, the forecasting trend curve follows the original data as a whole, and only a certain deviation occurs in some places where the trend jump is large.

**Figure 6:**
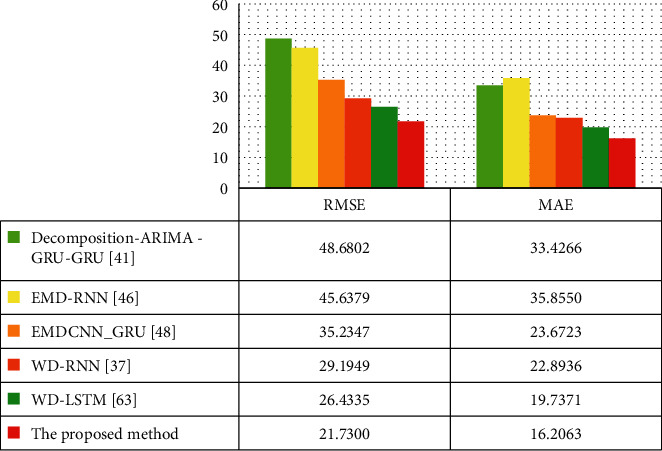
The RMSE and MAE of hourly PM2.5 in Beijing from March 22, 2016, to April 9, 2016, by decomposition-ARIMA -GRU-GRU [38], EMD-RNN [43], EMDCNN_GRU [45], WD-RNN [34], WD-LSTM [61], and the proposed method.

**Figure 7:**
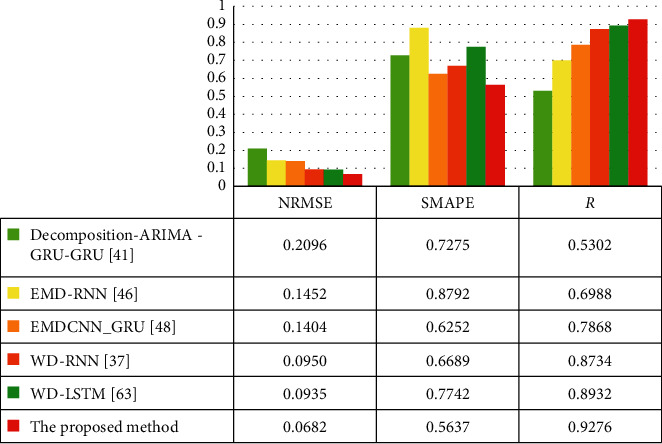
The NRMSE, SMAPE, and R of hourly PM2.5 in Beijing from March 22, 2016, to April 9, 2016, by decomposition-ARIMA -GRU-GRU [38], EMD-RNN [43], EMDCNN_GRU [45], WD-RNN [34], WD-LSTM [61], and the proposed method.

Table 5 gives the five evaluation indicators; the red value in the table is the optimal value of each indicator. Figures 6 and 7 show various indicators in the form of a histogram.

The WD-GRU model's evaluation indicators are the optimal values, among which the RMSE reaches 21.7300*μg*/m^3^. Compared with the EMDCNN_GRU [45] model based on the EMD decomposition method proposed in our previous study, the five indicators of RMSE, MAE, NRMSE, SMAPE, and *R* are improved by 38.3%, 31.5%, 51.4%, 9.8%, and 17.9%. The WD-GRU model has made significant progress with accuracy.

The experiments also verify the method selection of the combined model. For the combined model, the WD, EMD, and STL decomposition methods are used to decompose the PM2.5 sequence to reduce the complexity of the PM2.5 data, and then the RNN or GRU is used for forecasting. As to WD-RNN [34], EMD-RNN [43] in Table 5, the WD-RNN [34] model outperforms in all indicators. Compared with EMD-RNN [43], although both models use the same RNN network as a submodel, the WD-RNN [34] based on wavelet decomposition improved RMSE by 36.0%, MAE by 36.1%, NRMSE by 34.6%, SMAPE by 23.9%, and *R* by 25.0%. So we can find out that the wavelet decomposition method will have a good effect on the PM2.5 complex time series.

Simultaneously, we chose the GRU model as the submodel, which proved the right choice through experiments. The structure of WD-RNN [34], WD-LSTM [61], and the proposed WD-GRU model in Table 5 differ only in the selection of submodels. However, we see that the GRU network's proposed model as a submodel performs better in various indicators. Compared with the WD-LSTM [61] model, the RMSE of the proposed model is reduced by 4.7035 *μg*/m^3^. *R* increased from 0.8932 to 0.9276. Similarly, in the EMD-RNN [43] and EMDCNN_GRU [45] models, the effect of the model using the GRU network is also much better.

The wavelet decomposition and GRU credit that the improvement of the proposed model's indicators and the selection of hyperparameters during training play a decisive role in the performance of the resulting model. We use SMBO to determine the hyperparameters of the proposed model. These hyperparameters can develop the performance of the model effectively. The data in Table 5 shows that the WD-LSTM [61] model does not use SMBO to determine the hyperparameters. Its NRMSE is 0.0935*μg*/m^3^. The NRMSE of the proposed model is 0.0682*μg*/m^3^. Therefore, we can conclude that the degree of improvement is due to the replacement of the submodel with the GRU model and the SMBO method's credit.

In summary, our proposed WD-GRU model has a reasonably good effect on the multisteps forecasting task of PM2.5 concentration in the atmosphere per hour with a period of 24 hours.

## 5. Conclusions

This paper proposes a model combining wavelet decomposition and GRU network, in which wavelet decomposition is used to put down the complexity of the series time data. Then the GRUs are used to obtain component forecasting separately and finally achieve results through fusion. The Bayesian optimization is used to optimize each submodel's hyperparameters, wavelet decomposition layers, and mother wavelet function.

Experiments have confirmed that, in the multisteps forecasting of PM2.5 with 24 hours ahead, the model has an excellent performance. It is worth noting that the model we proposed is applicable not only in PM2.5 sequences but also in many similar data-driven forecasting tasks, such as temperature and humidity forecasting.

## Figures and Tables

**Algorithm 1:**
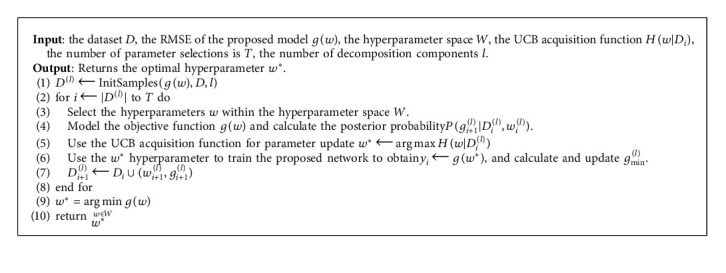
SMBO algorithm.

**Table 1:** Bayesian optimization hyperparameter space.

Hyperparameters	Type	Min	Max
Wavelet decomposition layers	Integer	1	15
Mother wavelet function	Categorical	{sym2, sym7, sym12, sym18,coif1, coif5, coif10, coif15, bior1.3, bior2.6, bior3.5, bior6.8, db3, db9, db13, db18, db35, db25, rbio1.1, rbio2.6, rbio3.5, rbio5.5, rbio4.4, rbio6.8'}	
No. 1 hidden units	Integer	{24,36,48}	
Dropout rate	Uniform	0	0.5
Batch-size	Integer	{1, 5, 10, 15, 20, 30, 50}	
Epochs	Integer	{100, 150, 200, 250, 300, 350, 400, 450, 500}	
Optimizer	Categorical	{Adadelta, Adam, Sgd}	

**Table 2:** Hyperparameters selected by Bayesian optimization.

Hyperparameters	Type	Bayesian optimization	Random search
Wavelet decomposition layer	Integer	8	10
Mother wavelet function	Categorical	db35	Sym7
No. 1 hidden units	Integer	48	24
Dropout rate	Uniform	0.0687	0
Batch-size	Integer	5	1
Epochs	Integer	350	300
Optimizer	Categorical	Adadelta	Adam

**Table 3:** Analysis of forecasting performance under different wavelet decomposition layers.

Combination mode	Number of levels	Number of GRUs	RMSE *μg*/m^3^	MAE *μg*/m^3^	NRMSE *μg*/m^3^	SMAPE	R
Mode no. 1	1	2	48.5712	32.8852	0.2176	0.7120	0.5361
Mode no. 2	2	3	48.9235	31.9738	0.1993	0.6746	0.5433
Mode no. 3	3	4	50.5702	32.0177	0.1675	0.6833	0.5562
Mode no. 4	4	5	48.2256	31.9763	0.1450	0.7128	0.6823
Mode no. 5	5	6	30.5029	24.0812	0.0991	0.6417	0.9086
Mode no. 6	6	7	22.0185	16.9521	0.0732	0.5773	0.9311
Mode no. 7	7	8	21.7539	16.3492	0.0704	0.5553	0.9270
Mode no. 8	8	9	21.7300	16.2063	0.0682	0.5637	0.9276
Mode no. 9	9	10	21.7168	16.6134	0.0703	0.5737	0.9329
Mode no. 10	10	11	22.1336	16.9992	0.0717	0.5802	0.9283

**Table 4:** Performance of models with different hyperparameter.

Hyperparameter optimization method	RMSE *μg*/m^3^	MAE *μg*/m^3^	NRMSE *μg*/m^3^	SMAPE	*R*
SMBO	21.7300	16.2063	0.0682	0.5637	0.9276
Random search	26.6429	18.7716	0.0888	0.5986	0.8926

**Table 5:** Indicators of each model in the same test set.

Model	RMSE *μg*/m^3^	MAE *μg*/m^3^	NRMSE *μg*/m^3^	SMAPE	*R*
Decomposition-ARIMA-GRU-GRU [40]	48.6802	33.4266	0.2096	0.7275	0.5302
EMD-RNN [45]	45.6379	35.8550	0.1452	0.8792	0.6988
EMDCNN_GRU [47]	35.2347	23.6723	0.1404	0.6252	0.7868
WD-RNN [36]	29.1949	22.8936	0.0950	0.6689	0.8734
WD-LSTM [72]	26.4335	19.7371	0.0935	0.7742	0.8932
The proposed method	21.7300	16.2063	0.0682	0.5637	0.9276

## Data Availability

Data used to support the findings of this study are available from the corresponding author upon request.
